# Molecular analysis of a new patient COX15 mutation provides insight into the etiology of fatal infantile cardioencephalopathy

**DOI:** 10.1016/j.jbc.2026.111475

**Published:** 2026-04-17

**Authors:** Jayda A. Carroll-Deaton, Iryna Bohovych, Faith T. Emetu, Jonathan V. Dietz, Elise D. Rivett, Elinor Stanley, Eric L. Hegg, Jennifer L. Fox, Oleh Khalimonchuk

**Affiliations:** 1 Department of Chemistry and Biochemistry, College of Charleston, Charleston, South Carolina, USA; 2 Department of Biochemistry, University of Nebraska, Lincoln, Nebraska, USA; 3 Department of Biochemistry and Molecular Biology, Michigan State University, East Lansing, Michigan, USA; 4 Nebraska Redox Biology Center, University of Nebraska, Lincoln, Nebraska, USA; 5 Fred & Pamela Buffett Cancer Center, Omaha, Nebraska, USA

**Keywords:** COX15, cytochrome *c* oxidase, heme, heme a synthase, mitochondria, yeast model

## Abstract

Mitochondrial disease can result from mutations in the enzymes responsible for biosynthesis of heme *a* and hemylation of respiratory complex IV of the electron transport chain, also known as cytochrome *c* oxidase (CcO). One of these enzymes, which is essential for assembly and function of CcO and thus function of the electron transport chain, is heme *a* synthase, COX15. A previously unknown fatal missense mutation of COX15, c.232G > A (p.Gly78Arg), was recently described in a case report by Galvão de Oliveira *et al.* Here, we show that the p.Gly78Arg-mimicking substitution in the homologous Cox15 protein in *Saccharomyces cerevisiae* (Gly95Arg) causes Cox15 protein instability and recapitulates the CcO defect observed in the patient. We demonstrate that the CcO defect observed with this Cox15 variant stems from insufficient heme *a* synthesis, and consequently, insufficient CcO hemylation and decreased levels of CcO. Our results provide insights into the etiology of the disease caused by this variant, suggesting that Cox15 protein instability and consequent attenuation of heme *a* synthase function is the main molecular factor behind the resulting multisystemic mitochondrial disorder in humans.

Mitochondrial oxidative phosphorylation (OXPHOS) diseases represent a major group of genetic disorders in humans, usually presenting in infants. One prominent facet of OXPHOS pediatric diseases stems from inborn errors in the biogenesis of respiratory complex IV, also known as cytochrome *c* oxidase (CcO) (1, 2). CcO is a large, multi-subunit enzyme of the mitochondrial electron transport chain (ETC) harboring several metallocofactors, most notably copper and heme *a*, the modified iron-protoporphyrin IX uniquely used by CcO (3, 4, 5). Two heme *a* molecules with different coordination geometries, designated heme *a* and heme *a*_3,_ reside in the Cox1 core subunit of CcO and are essential for both the stable folding of Cox1 and the catalytic function of CcO (3, 4). CcO biogenesis within the cell is a complex, multistep process that involves nearly 30 auxiliary factors, reviewed in (4, 5, 6), including those involved in the hemylation of the enzyme. Importantly, a number of fatal pediatric multisystemic diseases have been linked to mutations in these hemylation-associated factors (7, 8, 9, 10, 11, 12, 13, 14, 15, 16, 17, 18, 19, 20, 21).

Studies in yeast and mammalian cell culture models have established that heme *a* is synthesized through the actions of the enzymes of the heme *b* biosynthetic pathway followed by two modifying enzymes, Cox10 and Cox15, which are evolutionarily conserved, integral membrane proteins. The heme prenyltransferase, Cox10, mediates hydroxyethylfarnesylation of a specific heme *b* vinyl group, yielding the heme *o* intermediate. This intermediate is then converted to heme *a* by heme *a* synthase, Cox15, which oxidizes a particular heme methyl group to an aldehyde (22, 23). The bacterial homologs of Cox10 and Cox15 interact to form a complex, suggesting the heme *o* intermediate could be directly channeled between them (24); however, Cox10 and Cox15 interaction has thus far not been observed in eukaryotes, and topology modeling shows the active sites of the eukaryotic enzymes face different sides of the inner mitochondrial membrane (4, 25). In eukaryotes, Cox15 exists in a separate oligomeric complex than Cox10 (26, 27, 28), which forms regardless of whether the monomers are catalytically active, and these oligomers appear to be required for enzymatic activity (27, 28, 29, 30). Cox15 oligomers are stabilized by the CcO assembly factor Pet117, which helps couple heme *a* synthesis to CcO maturation (29) and is associated with neuropediatric disease (31).

Some structural information is available for the bacterial ortholog of Cox15 (32, 33), but the overall mechanistic understanding of this enzyme and its related pathological mutations remains incomplete. Several key aspects of the mechanism by which Cox15 catalyzes oxidation of heme *o* to produce heme *a* have been uncovered. This redox reaction requires electrons from the enzymes ferredoxin and ferredoxin reductase (34, 35) and additionally requires molecular oxygen (36), which is not the source of the aldehyde oxygen atom, yet serves as an oxidant during the Cox15 mechanism (37). A conserved glutamate residue (E166 in yeast), proposed to be a key active site residue (32), is essential for Cox15 activity in eukaryotes (30) and may serve to stabilize the carbocation intermediate proposed to form from the methyl group during the mechanism. This glutamate residue lies in the N-terminal half of the enzyme, which is likely the substrate-binding site for heme *o*, while the C-terminal half appears to bind a heme *b* cofactor (30, 32). Four conserved histidine residues of Cox15 (H169, H245, H368, and H431 in yeast) are essential for activity of the eukaryotic enzyme (28) and are proposed to serve as ligands to the iron center of the heme *o* substrate (H169 and H245) and the heme *b* cofactor (H368 and H431).

The mechanism by which heme *a* is installed into Cox1 during CcO biogenesis and whether Cox15 plays any role in this process is unknown. Two additional proteins, Shy1 (human SURF1) and Coa2 (which has no identified human counterpart), have been implicated in this step, but their roles are unclear (26, 27, 38, 39). Although heme *a* is installed in Cox1 during an early stage of CcO maturation, Cox15 associates with both fully assembled respiratory supercomplexes (containing CcO and complex III) and the complex III subunit Cor1 even in the absence of supercomplexes, potentially suggesting a role for Cox15 beyond heme *o* oxidation (40).

These missing details of Cox1 hemylation represent a significant gap in knowledge since mutations in conserved residues of COX15, COX10, and SURF1 in humans manifest in a variety of fatal pediatric diseases including tubulopathy and leukodystrophy (20), sensorineural deafness (8), fatal infantile hypertrophic cardiomyopathy (7, 8, 9), Charcot-Marie-Tooth disease type 1A (17), and Leigh Syndrome (10, 11, 15, 16, 18, 19, 21). Among these, infantile hypertrophic cardiomyopathy and Leigh Syndrome appear to be the predominant clinical manifestations. Consistent with these observations in patients, conditional skeletal muscle-specific Cox10 or Cox15 knockout mice demonstrate a CcO-deficient phenotype that resembles human mitochondrial myopathies (12, 14).

The most recently reported patient mutation in COX15 is the missense mutation c.232 G > A (p.Gly78Arg) (13). This mutation was compound heterozygous with a nonsense c.452 C > G (p.Ser151X) mutation in a male pediatric patient afflicted with hypertrophic cardiomyopathy, hyperlacticaemia, and CcO deficiency, which falls into the category of a mitochondrial complex IV deficiency nuclear type 6 disorder (MC4DN6; OMIM accession number 615119) (13). Since the allele resulting in p.Ser151X is known to cause loss of function and has previously been shown to cause mitochondrial disease when it is compound heterozygous with a missense mutation (7, 10), the remaining question is why the newly identified missense mutation resulting in G78R substitution is unable to yield a functional protein. In the present study, we used the genetically tractable *Saccharomyces cerevisiae* eukaryotic model to carry out an in-depth molecular analysis of the G78R variant of COX15 (G95R in yeast Cox15) and determined how this amino acid substitution leads to mitochondrial disease.

## Results

### The MC4DN6-causing amino acid substitution results in low Cox15 protein levels

The clinical study by Galvão de Oliveira *et al.* (13) that documented the p.G78R patient variant did not include an in-depth biochemical study of the patient-derived cells. Motivated to better understand the pathological mechanism by which the p.G78R substitution leads to MC4DN6, we therefore analyzed this substitution in a yeast genetic model. Cox15 is highly conserved, and it has been shown that human *COX15* is able to complement the yeast *cox15*Δ deletion mutant (41). Thus, it is generally established that yeast Cox15 can be used as a reliable model to gain insight into the human enzyme (22, 28).

**Figure 1:**
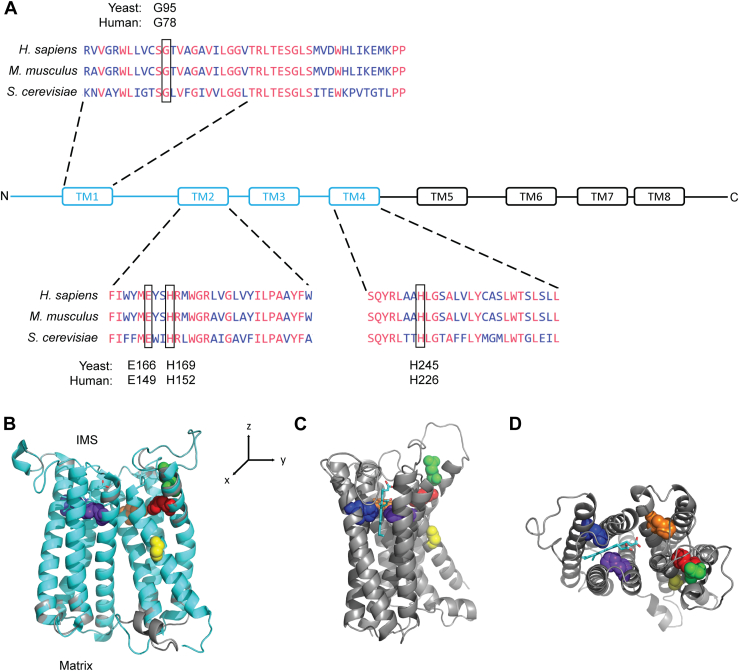
**Location and conservation of the Gly-78 (Gly-95 in yeast) amino acid residue of Cox15.***A*, multiple alignment of human COX15 isoform 1, mouse Cox15, and yeast Cox15, showing selected conserved regions in the N terminal half of the protein (*lightblue* in the schematic). Specifically, sequences are shown for transmembrane (TM) helices TM1, TM2, and TM4, which contain the conserved residues G95, E166, H169, and H245 in yeast (corresponding to G78, E149, H152, and H226, respectively, in humans), as well as a conserved loop shielding the proposed active site in the region linking TM1 and TM2. Residues in *pink* are identical in all three species, while residues in *blue* are either conservatively substituted or less conserved. *B*, model produced by one-to-one threading of yeast Cox15 protein (*gray*) (lacking the predicted mitochondrial targeting sequence) onto the crystal structure for heme, *a* synthase in *Bacillus subtilis* (PDB 6A2J, *light blue*), showing the predicted location of G95 (*yellow*), along with the locations of four conserved histidine residues (H169 in *red*, H245 in *orange*, H368 in *darkblue*, and H431 in *purple*), the heme *b* cofactor (*light blue sticks*), and the proposed catalytic residue E166 (*green*). The individual amino acid residues indicated above are represented by space-filling spheres instead of the ribbon model. Regions where the yeast protein backbone does not fit perfectly onto the bacterial homolog crystal structure appear *gray*. IMS, intermembrane space. *C*, different viewing angle of the model in B produced by approximately 90° rotation about the *z* axis, with the bacterial homolog removed for clarity. *D*, different viewing angle of the model in B produced by approximately 90° rotation about the *y* axis, with the bacterial homolog removed for clarity. PDB, Protein Data Bank.

A comparison of amino acid sequences of human and mouse COX15 with their yeast ortholog demonstrates a significant degree of conservation (the yeast ortholog shares 39% identity with the mouse ortholog and 41% identity with the human ortholog; Figure 1*A*). Importantly, the glycine residue substituted for arginine in the p.G78R patient variant is conserved in yeast (G95, marked with a box in Fig. 1*A*), so the G95R variant in yeast is analogous to the patient variant. Structural modeling of yeast Cox15 based on homology to the recently solved crystal structure of the *Bacillus subtilis* heme *a* synthase ortholog CtaA (32) reveals that G95 is in the middle of the first transmembrane alpha helix of the N-terminal domain of the protein (Fig. 1, *B*–*D*), which is the domain proposed to bind heme *o* substrate (32).

**Figure 2:**
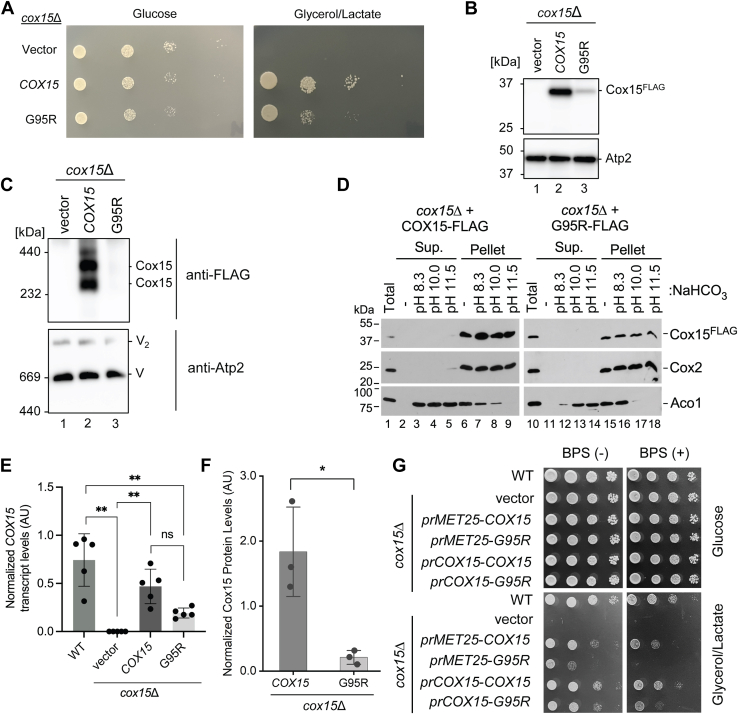
**The G95R Cox15 variant has a respiratory growth defect, lower steady-state levels of Cox15, and lower levels of Cox15 oligomeric complexes, compared to wildtype.***A*, respiratory growth test at 30 °C of *cox15*∆ cells expressing plasmid-borne WT Cox15-FLAG (*COX15*), the G95R Cox15-FLAG variant (G95R), or vector control (Vector) from the *MET25* promoter. Cells were cultured overnight in 2% glucose-containing synthetic medium lacking uracil for plasmid selection. Aqueous solutions of these cells normalized to *A*_600_ = 1 were serially diluted onto synthetic medium plates lacking uracil and containing either 2% glucose or 2% glycerol/lactate. Results are shown for one experiment, representative of five independent experiments (with ten total biological replicates). *B*, steady-state levels of Cox15 WT and G95R variant proteins with loading control (Atp2, the β subunit of the F_1_ domain of mitochondrial complex V) in mitochondria of cells described in A, analyzed by SDS-PAGE with immunoblotting using anti-FLAG and anti-Atp2 antibodies, respectively. Note that the anti-Atp2 immunoblot images in Fig. 2B and Fig. 3G are identical because those figures are from the same gel and therefore the same membrane; we are focusing on the anti-FLAG detection in Fig. 2B, with anti-Atp2 shown as the loading control. The positions of molecular mass markers are indicated on the left side of the immunoblots. Results are shown for one experiment, representative of three independent experiments (biological replicates). *C*, BN-PAGE analysis of Cox15 oligomeric complexes and mitochondrial complex V (loading control) in mitochondria of cells described in A, visualized by immunoblotting for FLAG and Atp2, respectively. Note that the anti-Atp2 immunoblot images in Fig. 2C and Fig. 3F are identical because those figures are from the same gel and therefore the same membrane; we are focusing on the anti-FLAG detection in Fig. 2C, with anti-Atp2 shown as the loading control. The positions of molecular mass markers are indicated on the *left* side of the immunoblots. Results are shown for one experiment, representative of three independent experiments (biological replicates). *D*, alkaline extraction of proteins from mitochondria of *cox15*∆ cells expressing either plasmid-borne WT Cox15-FLAG or the G95R Cox15-FLAG variant from the *MET25* promoter. Samples were subjected to the indicated alkaline conditions or untreated (-) for 60 min on ice and fractionated at 65,000*g* for 1 h at 4 °C. Fractions were analyzed by SDS-PAGE with immunoblotting using anti-FLAG, anti-Cox2, and anti-aconitase (Aco1) antibodies. The positions of molecular mass markers are indicated on the left side of the immunoblots. Results are shown for one experiment, representative of three independent experiments (biological replicates). *E*, levels of *COX15* mRNA transcripts for WT cells and the cells described in Figure 2A. Transcript levels were measured by the ∆∆C_T_ method and normalized to b-actin expression levels; results are shown for five biological replicates. ∗∗Significant differences by one-way ANOVA with a post hoc Tukey’s comparison test (p<0.01); ns = not significantly different. AU, arbitrary units. *F*, levels of Cox15 protein for *cox15*∆ cells expressing plasmid-borne WT Cox15-FLAG (*COX15*) and the G95R Cox15-FLAG variant (G95R). Protein levels were assessed by band quantification of immunoblot images, and the anti-FLAG signal in each lane was normalized to the signal of the loading control (anti-porin or anti-Atp2); results are shown for three biological replicates. ∗Significant difference by an unpaired *t* test (*p* < 0.02). *G*, respiratory growth test at 30 °C for WT cells and the cells described in Figure 2A along with *cox15*∆ cells expressing *COX15* or G95R variant on the high-copy YEp plasmid from the endogenous *COX15* promoter. Cells were cultured overnight in 2% glucose-containing synthetic medium lacking uracil for plasmid selection of the transformants or similar medium containing uracil for WT. Aqueous solutions of these cells normalized to *A*_600_ = 1 were serially diluted onto synthetic medium plates containing either 2% glucose or 2% glycerol/lactate, with (+) or without (-) 100 μM bathophenanthroline disulfonate (BPS). Results are shown for one experiment, representative of three independent experiments (with three total biological replicates). BN, blue-native.

We generated the p.G78R-mimicking G95R variant of yeast Cox15 on a plasmid and expressed it in the *cox15*Δ yeast deletion strain. This variant has a C-terminal FLAG epitope tag, which we previously showed does not interfere with the protein’s stability or function (28, 29). We found the G95R variant of Cox15-FLAG is unable to support the growth of the *cox15*Δ strain on a non-fermentable carbon source (glycerol/lactate) as robustly as the corresponding WT Cox15-FLAG protein (Figure 2*A*). Interestingly, this growth defect was only observed with the FLAG-tagged protein (but not a 13xMyc-tagged protein), and the growth defect was only observed when cells were precultured in glucose (but not galactose) prior to the growth test (data not shown). Consistent with the latter observation, we found the growth of cells cultured in 2% galactose with 0.1% glucose (a nonrepressive condition which permits respiration) was significantly slower for cells expressing the G95R variant of Cox15 than those expressing the WT protein (Fig. S1), reflecting a respiratory deficit. Although it is tempting to speculate that the FLAG epitope contributes to some distortion in the protein structure, additional studies would be needed to confirm this potential explanation.

Finding conditions under which a respiratory defect could be observed for cells expressing Cox15 with the G95R substitution compared to an isogenic WT Cox15, which is consistent with the patient phenotype, allowed us to use the yeast model to characterize the effects of the p.G78R (G95R) substitution. We also tested whether the G95R variant caused a dominant negative effect when overexpressed in WT and found no appreciable effect (data not shown). In addition, we tested growth of cells expressing Cox15 with the G95R substitution compared to the WT Cox15, under the control of the native promoter and using low-copy-number YCp plasmids. Results under these conditions were consistent with results using a high-copy-number YEp plasmid with expression from the *MET25* promoter (Fig. S2).

We isolated mitochondria from *cox15*Δ cells expressing the G95R variant, the WT Cox15 protein, or vector control. Steady-state levels of the G95R variant in the *cox15*Δ strain are markedly decreased compared to the WT protein (Figs. 2*B* and S3). This lower level of Cox15 protein results in a drastic reduction in the levels of Cox15 oligomeric complexes, as revealed by blue-native (BN)-PAGE with anti-FLAG immunoblotting (Fig. 2*C*). This finding is significant because our previous work suggests the oligomeric complexes formed by Cox15 are important for heme *a* synthase activity (28, 29). We assessed the membrane extractability of these Cox15 complexes and found it is unchanged in the G95R variant (Fig. 2*D*).

The low levels of Cox15 observed for the G95R variant could result from either reduced expression or increased proteolysis. In the experiments described above, the G95R and WT Cox15 proteins are expressed from identical plasmids (which are high-copy plasmids expressing the protein under the control of a heterologous promoter from the *MET25* gene). Therefore, equivalent amounts of G95R and WT Cox15 protein are expected to be expressed. To test this, we measured both the levels of mRNA transcripts by quantitative real-time PCR (qPCR) and the levels of Cox15 protein by western blot band quantification (for both the WT Cox15 protein and the G95R variant). Analysis of mRNA transcripts by qPCR showed no significant difference in the abundance of transcripts encoding the G95R variant and the WT Cox15 protein expressed in *cox15*Δ cells (Fig. 2*E*). The mean transcript level for the G95R variant is 2.4 times lower than the mean transcript level for WT Cox15, with no significant difference between them. In contrast, the mean protein abundance for the G95R variant is 8.6 times lower than the mean protein abundance of WT Cox15, which was significantly different (*p* < 0.02) (Fig. 2*F*). Therefore, these results suggest the G95R variant of Cox15 is inherently unstable and low steady-state levels of protein are detected because the unstable protein is degraded by the cell's quality control mechanisms. We tested several candidate protein degradation mechanisms, including the ubiquitin-proteasome system and the topologically relevant inner mitochondrial membrane proteases, Yme1, Pcp1, and Oma1. However, inhibition of these enzymes or deletion of the genes encoding them had no appreciable effect on the stability of the G95R variant, indicating that none of these mechanisms is responsible for its degradation (data not shown).

We additionally observed a significant respiratory growth defect of the G95R variant in the presence of the iron chelator bathophenanthroline disulfonate (BPS), which was observed when the proteins were expressed from either the *MET25* promoter from a high-copy vector (Fig. 2*G*) or the endogenous *COX15* promoter from either high- or low-copy vectors (Figs. 2*G* and S2). This growth defect was more severe than that observed in the absence of BPS, suggesting under respiratory conditions when the ETC is required and therefore functional iron-containing hemes are required, cells expressing the G95R variant are impaired in the ability to perform OXPHOS. Therefore, we performed further experiments described below to investigate mitochondrial hemes, ETC activity, and ETC complexes in the G95R variant.

### Mitochondria from cells expressing the G95R variant exhibit a CcO defect

**Figure 3:**
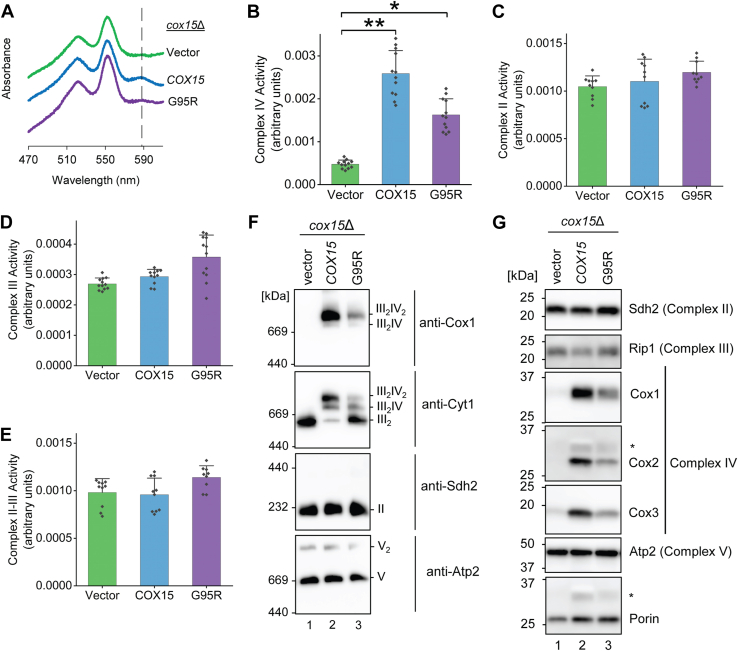
**Mitochondria from cells expressing the G95R Cox15 variant have decreased levels of fully assembled and hemylated cytochrome c oxidase.***A*, heme-pyridine redox difference spectra of mitochondria from the cells described in Figure 2A, cultured in 2% galactose and 0.1% glucose synthetic medium lacking uracil for plasmid selection. Spectra are offset for clarity, and a *dashed line* is drawn to indicate the heme *a* absorption maximum. Results are shown for one experiment, representative of three independent experiments (biological replicates). *B*-*E*, enzymatic activity in the mitochondria described above determined by spectrophotometric assays for (B) complex IV (CcO) (Cox15 and vector control are significantly different from each other with ∗∗*p* < 0.01, and G95R and vector control are significantly different from each other, ∗*p* < 0.05, by one-way ANOVA with Tukey HSD post hoc test), (C) complex II (succinate dehydrogenase), (D) complex III (the cytochrome *bc*_1_ complex), and (E) combined complex II-III. *Bars* indicate the average and S.D. of 3 biological replicates, each tested with 3-5 technical replicates (shown superimposed on the bars). *F*, BN-PAGE analysis of respiratory complexes in the mitochondria described above. Complexes II, III, IV, and V (and supercomplexes containing complexes III and IV) were visualized by immunoblotting for Sdh2, Cyt1, Cox1, and Atp2, respectively, with complex V serving as the loading control. Note that the anti-Atp2 immunoblot images in Fig. 2C and Fig. 3F are identical because those figures are from the same gel and therefore the same membrane; we are focusing on the anti-Cox1, anti-Cyt1, and anti-Sdh2 detections in Fig. 3F, with anti-Atp2 shown as the loading control. The positions of molecular mass markers are indicated on the left side of the immunoblots. Results are shown for one experiment, representative of three independent experiments (biological replicates). *G*, steady-state levels of representative subunits of respiratory complexes II (Sdh2), III (Rip1), IV (Cox1, Cox2, and Cox3), and V (Atp2), with loading control (Porin) in the mitochondria described above, analyzed by SDS-PAGE with immunoblotting. Note that the anti-Atp2 immunoblot images in Fig. 2B and Fig. 3G are identical because those figures are from the same gel and therefore the same membrane. The positions of molecular mass markers are indicated on the *left* side of the immunoblots. *Asterisks* indicate faintly visible bands from previous detections of other proteins on the same membrane. Results are shown for one experiment, representative of three independent experiments (biological replicates). BN, blue-native; CcO, cytochrome c oxidase.

To systematically characterize the molecular underpinnings of the G95R variant, we isolated and analyzed mitochondria from *cox15*Δ cells expressing the G95R variant. These mitochondria exhibit decreased, yet detectable, levels of heme *a* compared to the Cox15 WT control, as determined by heme redox difference spectroscopy (Figure 3*A*). This result is consistent with the observed impairment in respiratory growth and additionally suggests a CcO-specific defect is the cause of that respiratory growth impairment.

We next evaluated enzymatic activities of the yeast ETC complexes. Mitochondria from *cox15*Δ cells expressing the G95R variant display a decrease in CcO activity compared to the WT Cox15 protein (Fig. 3*B*), although this difference was not statistically significant, whereas the activities of respiratory complexes II and III as well as combined complex II-complex III activity remain largely unchanged (Fig. 3, *C*–*E*). Note that the *cox15*Δ cells expressing vector control do not have any CcO activity, and the low level shown in the graph represents the steady background oxidation rate of reduced cytochrome *c* by oxygen in the air. There was also some indication that complex III activity is slightly elevated in cells expressing the G95R variant, which has been observed previously for cells impaired in CcO activity (42, 43).

BN-PAGE immunoblotting of respiratory complexes from G95R-expressing mitochondria revealed that the OXPHOS complexes II (anti-Sdh2 blot) and V (anti-Atp2 blot) are present at levels comparable to the WT control (Fig. 3*F*). However, the complex IV-containing supercomplexes in which one or two copies of complex IV associate with dimeric complex III are reduced in concentration (anti-Cox1 and anti-Cyt1 blots) and, consequently, there is an accumulation of unincorporated complex III dimer (anti-Cyt1 blot) (Fig. 3*F*). Consistent with this result, steady-state levels of the core CcO subunits (Cox1, Cox2, and Cox3), but not representative subunits of the other OXPHOS complexes, are present at lower concentrations in mitochondria from cells expressing the G95R variant (Fig. 3*G*).

Collectively, these results demonstrate that the defect associated with the G95R conversion in Cox15 leads to CcO deficiency due to a decreased level of fully assembled and hemylated CcO and suggest the mutation causing the human G78R and yeast G95R substitution results in loss of protein and therefore reduced ability to synthesize heme *a*.

### The G95R variant produces an insufficient amount of heme a

The results above suggest the G95R amino acid conversion reduces the stability of the Cox15 protein, leading to lower levels of enzyme available to produce heme *a*, thus impairing the hemylation of Cox1 during CcO assembly and resulting in CcO deficiency. To support this interpretation, we 1) evaluated the complexes formed by CcO assembly factors and by Cox1-containing CcO assembly intermediates that are formed prior to and after Cox1 hemylation (4, 27, 38), 2) looked for any formation of prooxidant heme-containing assembly intermediates, and 3) quantified mitochondrial hemes by HPLC.

**Figure 4:**
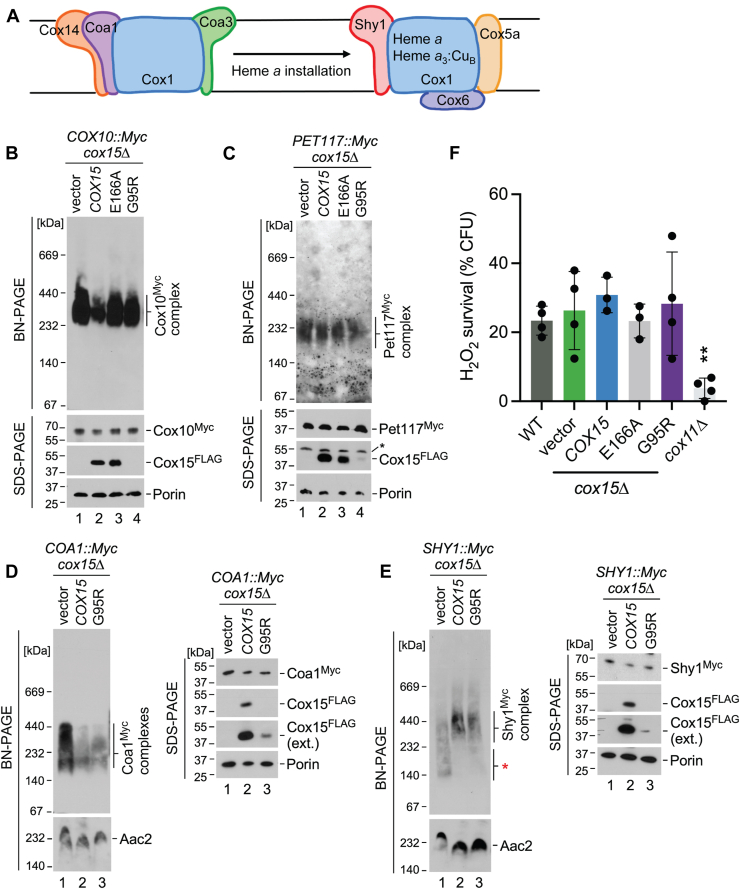
**Mitochondria from cells expressing the G95R Cox15 variant exhibit a partial defect in the Cox1 hemylation stage of cytochrome c oxidase assembly.***A*, schematic of the assembly intermediates that can be isolated before and after heme *a* installation into Cox1 during CcO biogenesis, showing the assembly factors and CcO subunits that have been identified in each complex. The first assembly intermediate depicted is characterized by the presence of Coa1, while the second one is characterized by the presence of Shy1 and the heme *a* cofactors within Cox1. *B*-*E*, BN-PAGE analysis of mitochondrial lysates from *cox15*∆ cells expressing plasmid-borne WT Cox15-FLAG (*COX15*), the G95R Cox15-FLAG variant (G95R), the E166A catalytic variant, or vector control (Vector), showing B) Cox10 complexes, (C) Pet117 complexes, (D) the CcO assembly intermediate characterized by the presence of Coa1, and (E) the CcO assembly intermediate characterized by the presence of Shy1, each assessed in a strain expressing a Myc-tagged version of those proteins. Cells were cultured in selective synthetic medium with 2% glucose. SDS-PAGE analysis of the same samples is shown below or to the right of each corresponding BN-PAGE immunoblot. Labeled proteins were detected by immunoblotting with appropriate antibodies; porin and the mitochondrial IM ADP/ATP carrier Aac2 were used as the loading controls for SDS-PAGE and BN-PAGE, respectively. The positions of molecular mass markers are indicated on the *left* side of the immunoblots. The bands marked with an *asterisk* in (C) (Cox15-FLAG immunoblot) represent nonspecific antibody binding. The Cox15-FLAG (ext.) immunoblot shown in (D) is an additional image of the Cox15-FLAG immunoblot after a longer exposure time. The *red asterisk* in (E) indicates the lower molecular weight Shy1 complexes observed in cells expressing vector control and the G95R variant. Results are shown for one experiment, representative of three independent experiments (biological replicates). *F*, cell viability after acute 1 mM hydrogen peroxide challenge for 1 h at 30 °C measured as the number of viable colony-forming units after treatment normalized to the number of viable colony-forming units resulting from untreated cells, from at least three biological replicates. Cells were cultured in 2% glucose. The only sample that was significantly different from WT was the *cox11*Δ control. ∗∗Significant difference by one-way ANOVA with a post hoc Tukey’s comparison test (*p* < 0.05). BN, blue-native; CcO, cytochrome c oxidase.

Many assembly factors are required in the early stages of CcO biogenesis that lead to heme *a* installation into Cox1 (4, 5, 6). The assembly intermediate complexes formed by the newly synthesized Cox1 before and after heme *a* installation are shown in Figure 4*A*. This heme *a* synthesis and Cox1 hemylation step requires the CcO assembly factors Cox10, Cox15, and Pet117 (and potentially others).

BN-PAGE analysis of mitochondrial lysates from *cox15*Δ cells with Cox10-Myc tagged protein or Pet117-Myc tagged protein expressing either vector control, WT Cox15, the E166A catalytic variant, or the G95R variant revealed that complexes formed by Cox10 and by Pet117 were unchanged in the G95R variant (Fig. 4, *B* and *C*). BN-PAGE analysis of Coa1-Myc tagged protein from mitochondrial lysates showed that the CcO assembly intermediate formed prior to heme *a* installation into Cox1 (which contains the assembly factor Coa1) is also similar in the G95R variant compared to WT, while cells expressing empty vector show an accumulation of this complex (Fig. 4*D*). In contrast, the hemylated CcO assembly intermediate formed after heme *a* installation into Cox1 (which contains the assembly factor Shy1) is attenuated in mitochondrial lysates from *cox15*Δ cells expressing empty vector (Fig. 4*E*), reflecting impaired Cox1 hemylation in the absence of Cox15. Cells expressing the G95R variant showed a slight attenuation of this assembly intermediate and a small amount of the less massive Shy1-containing complex that is evident in *cox15*Δ cells expressing empty vector (Figure 4*E*, red asterisk), consistent with a partial defect at this stage of CcO assembly.

Since heme is an inherently toxic molecule capable of redox chemistry (23, 44), its faulty incorporation into target proteins such as CcO can lead to the formation of prooxidant heme-containing assembly intermediates (45, 46). Therefore, we examined whether the G95R Cox15 variant led to formation of any prooxidant complexes or otherwise disrupted the heme *a* biosynthetic pathway in a manner that promotes cytotoxic effects *via* an established quantitative functional assay (29, 45). This assay measures the survival rate of cells after an acute 1-h exposure to hydrogen peroxide. In contrast to other CcO assembly mutants such as *cox11*Δ that have been previously shown to exhibit marked sensitivity to exogenous hydrogen peroxide (45) (Fig. 4*F*), *cox15*Δ cells expressing the G95R Cox15 variant did not display any appreciable difference in their tolerance to acute hydrogen peroxide stress when compared to the corresponding WT cells (Fig. 4*F*). Consistent with this result, activity of the reactive oxygen species-sensitive mitochondrial enzyme aconitase in the G95R variant-expressing cells was unaltered relative to COX15-expressing cells (data not shown), suggesting a lack of any substantial oxidative damage in these cells.

**Figure 5:**
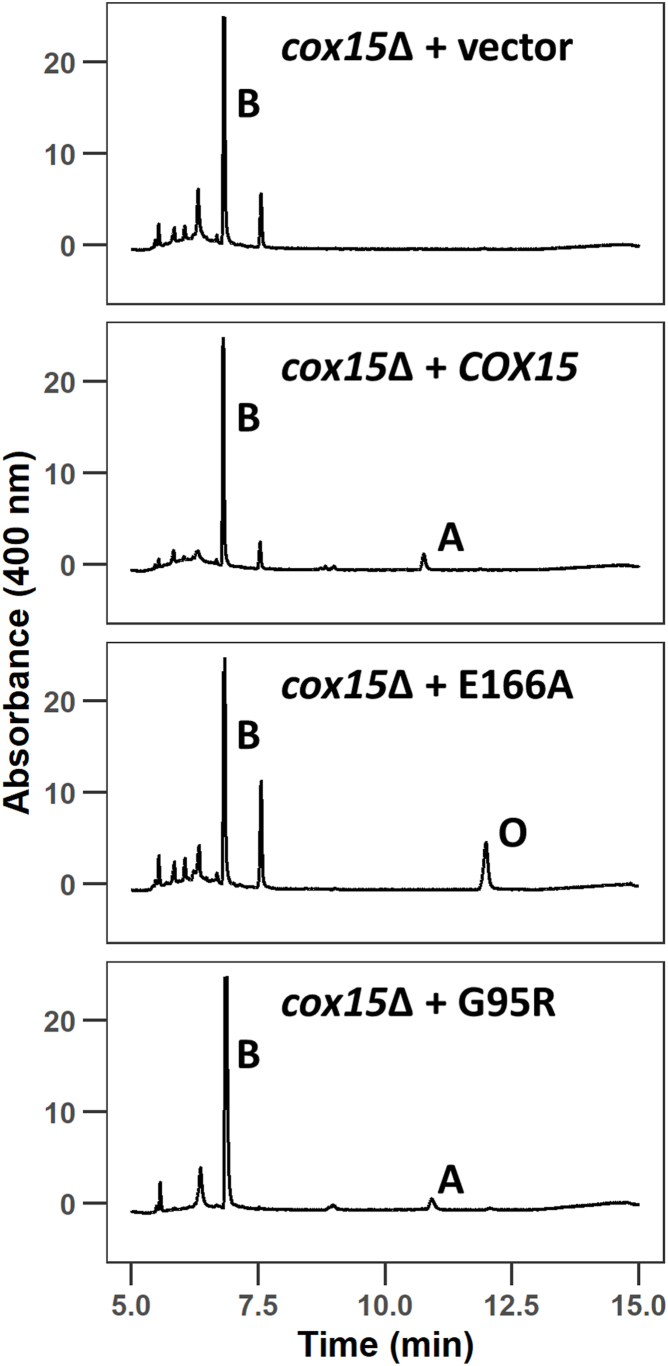
**Mitochondria from cells expressing the G95R Cox15 variant exhibit decreased levels of heme *a*.** HPLC chromatography was used to compare noncovalently bound hemes extracted from mitochondria. Mitochondria were isolated from *cox15*Δ cells expressing plasmid-borne vector control, WT Cox15-FLAG, E166A Cox15-FLAG variant (data previously published, (30)), or G95R Cox15-FLAG variant. Cells were cultured in 2% glucose-containing synthetic medium lacking uracil for plasmid selection. Chromatograms are shown for one experiment representative of 2 to 3 biological replicates. Heme *b* is quantified in each of these types of mitochondria based on a standard curve, and levels of heme *b*, heme *o*, and heme *a* are tabulated as a percentage of the total extractable heme for each sample in Table 1. HPLC, high-pressure liquid chromatography; WT, wildtype.

To assess the heme *a* synthase activity of the G95R variant, noncovalently bound mitochondrial hemes were quantified by HPLC from *cox15*Δ cells expressing vector control, WT Cox15, the G95R variant, or the E166A variant. We recently showed that the E166A variant does not affect Cox15 steady-state protein levels or the ability of Cox15 to oligomerize, but it does eliminate Cox15 catalytic activity (30). Consequently, mitochondria from E166A-expressing *cox15*Δ cells show no heme *a* peak and accumulate substantial amounts of the heme *o* biosynthetic precursor (Fig. 5; Table 1). In contrast, mitochondria from *cox15*Δ cells expressing vector control (and thus lacking all Cox15 protein) lack both heme *a* and heme *o*. Mitochondria from *cox15*Δ cells expressing the G95R variant have a heme species pattern similar to WT where heme *a* but not heme *o* is detectable; however, heme *a* levels are drastically reduced compared to WT (Fig. 5; Table 1).

**Table 1:** Noncovalently bound hemes extracted from mitochondria

Mitochondria	Heme *b* (μM)^a^	Heme *b* (%)^b^	Heme *o* (%)^b^	Heme *a* (%)^b^	*n*	Reference
*cox15Δ*+ vector	6.6 ± 1.7	100	ND	ND	2	Rivett, *et al.*, 2023 (30)
*cox15Δ*+ *COX15*	9.4 ± 4.7	87–95	ND	5–13	3	Rivett, *et al.*, 2023 (30)
*cox15Δ*+ E166 A	11.0 ± 2.8	78–88	12–22	ND	2	Rivett, *et al.*, 2023 (30)
*cox15Δ*+ G95 R	5.4 ± 0.6	93–94	ND	6–7	2	This study

a Average ± standard deviation for *n* biological replicates. Heme *b* was quantified based on a standard curve.

b Range for *n* biological replicates. For each replicate, the percentage of total extractable heme was determined using the area under each heme peak. ND = not determined due to levels falling below the limit of detection.

Altogether, these results strongly suggest the G95R amino acid residue conversion in Cox15 does not impair the enzyme’s catalytic activity but rather leads to protein instability, causing a substantial amount of the cell's Cox15 protein to be degraded. The remaining fraction of Cox15, while fully functional, is unable to supply maturing CcO with enough heme *a* to meet the cell's bioenergetic demands.

## Discussion

The heme *a* synthase, Cox15, is a highly conserved critical player in CcO biogenesis. To date, four missense mutations and one nonsense mutation in COX15 have been associated with MC4DN6 mitochondrial disorders that primarily manifest as neurological Leigh syndrome and fatal infantile cardiomyopathy (7, 9, 10, 13, 14). However, in many cases, the molecular etiology of these disorders remains understudied. Here, using the *S. cerevisiae* genetic model, we carried out a detailed molecular analysis of the most recently reported MC4DN6-associated COX15 mutation. Our data demonstrate the G95R variant in yeast Cox15 mimicking the p.G78R substitution in the human enzyme recapitulates the patient CcO defect, underscoring the utility of the yeast model to investigate the molecular basis of human pathogenic mutations.

Our results demonstrate the p.G78R-mimicking Cox15 variant is posttranslationally destabilized, but not completely destroyed, thereby yielding a functional, albeit significantly attenuated Cox15 oligomeric complex. Therefore, despite its inherent instability, the residual protein retains its ability to fold, assemble, and maintain essential interactions akin to the WT heme *a* synthase. Such a scenario is not unprecedented and has been reported previously for other mitochondrial enzymes (47).

Analysis of noncovalently bound mitochondrial hemes further indicates that, in contrast to the vector control and catalytic E166A variant control, *cox15*Δ cells expressing the G95R variant can generate markedly decreased but detectable amounts of heme *a*, without accumulation of the heme *o* biosynthetic precursor. That residual heme *a* species appears to become incorporated into the maturing CcO, yielding functional enzyme at severely attenuated concentrations. This interpretation is based on the evidence that the G95R variant-expressing cells are: (i) partially respiratory competent, (ii) retain partial CcO activity, and (iii) do not appear to harbor prooxidant CcO assembly intermediates as indicated by unaltered tolerance of cells to acute oxidative challenge. Consistent with the loss of Cox15 function, the CcO biogenesis defect is observed at the stage of Cox1 maturation that involves formation of the heme *a*-containing sites. Therefore, the protein instability caused by the G95R variant leads to a decreased concentration of functional Cox15, low resulting levels of its heme *a* product, and low resulting levels of CcO. Our findings provide new insights into the molecular etiology of fatal infantile cardioencephalopathy and suggest that mitochondria-targeted delivery of heme *a* or an emerging whole-mitochondria transplantation technology might represent a viable therapeutic strategy in MC4DN6 patients.

While baker's yeast has been established as a robust system to model human mitochondrial diseases (48, 49), an important question that remains to be addressed is whether the genetic and biochemical data obtained with our yeast model accurately reflect the situation in patients with this specific variant of COX15. For example, recent studies of metal incorporation into human CcO revealed surprising differences from the yeast enzyme (46). Even though the CcO hemylation process is believed to be largely conserved among eukaryotes, such a potential difference should not be dismissed. Validation of our findings in cultured human cells engineered to harbor the p.G78R variant remains an important future research pursuit.

## Experimental procedures

### Yeast reagents and culture

Yeast strains used in this study were previously described (28, 29, 38, 45). The high-copy plasmid encoding Cox15 with a C-terminal FLAG epitope tag expressed from the *MET25* promoter was previously described (28). The G95R variant was made from that plasmid by site-directed mutagenesis using the Q5 site-directed mutagenesis kit (New England BioLabs). Those two plasmids include the *URA3* gene as a selection marker, and *LEU2* versions were also created *via* homologous recombination to replace the *URA3* gene with *LEU2* as an alternate selection marker. Versions of the plasmids were also created with the *COX15* promoter by replacing the *MET25* promoter using homologous recombination. A YCp version of that plasmid was also created *via* homologous recombination to add the gene for *COX15* (WT or G95R variant) with its FLAG tag and the *COX15* promoter and *CYC1* terminator to an empty YCp plasmid. All plasmids were confirmed by DNA sequencing.

Cells were cultured and handled in accordance with published protocols (50), and cells were transformed with plasmids *via* the lithium acetate method (51). To maintain plasmid selection, unless specified otherwise, cells transformed with plasmids were cultured in Brent Supplement Mixture synthetic selective medium (Sunrise Science Products and U.S. Biological Life Sciences) containing either 2% galactose and 0.1% glucose (for mitochondrial isolation) or 2% glucose (for growth assays and HPLC analysis of mitochondrial heme levels), lacking uracil to maintain plasmid selection.

### Growth assays

Respiratory growth tests were performed at 30 °C as previously described (28). Cells were precultured overnight in synthetic selective medium containing 2% glucose then normalized to A_600_ = 1 in sterile water, serially diluted, and dropped onto synthetic selective medium plates containing 2% glucose or 2% glycerol/lactate as a carbon source. The growth was assessed and documented after 1 to 2 (glucose plates) or 2 to 5 (glycerol/lactate plates) days of incubation at 30 °C. Both the overnight cultures and the plates lacked uracil to maintain plasmid selection. Growth tests in the presence *versus* absence of BPS were performed similarly and at 30 °C, except the overnight culture of WT cells contained uracil, the plates contained uracil, and the indicated plates contained 100 μM BPS. Quantitative hydrogen peroxide sensitivity tests were also performed at 30 °C in accordance with the previously published protocol (52), and cells were cultured in 2% glucose. Growth curves were assessed by inoculating single colonies in 5 ml of selective synthetic medium with 2% glucose for overnight growth at 30 °C followed by washing, resuspending, and inoculation into 50 ml of prewarmed (30 °C) selective synthetic medium with 2% galactose and 0.1% glucose at a starting A_600_ of ∼0.05. After 8 h, the cellular growth at 30 °C was measured hourly for the duration of the next 16 h.

### Mitochondrial isolation and assays

Mitochondria were isolated from yeast cells by established protocols (53). Total mitochondrial protein concentration was determined by the Bradford method using the Coomassie Plus assay kit (Thermo Fisher Scientific). Heme-pyridine redox difference spectra were determined from SDS-solubilized mitochondrial lysates according to the method of Berry and Trumpower (54). Complex II, III, IV, and combined II-III activity of isolated mitochondria was assessed as previously described (55, 56). The y-axis is in arbitrary units of absorbance change over time, and data are shown for equivalent amounts of total mitochondrial protein, as measured by the Bradford assay. Statistical significance was determined by ANOVA with Tukey HSD *post hoc* test, using the means of the technical replicates performed on each biological replicate (n = 3 biological replicates per sample).

### HPLC analysis of mitochondrial heme levels

Noncovalently bound hemes were extracted from mitochondria as described previously (30). Briefly, 0.25 to 0.5 mg total protein was used in each extraction. After pelleting the appropriate amount of mitochondria and resuspending in a small volume of 600 mM sorbitol, 20 mM Hepes, pH 7.4 buffer (25–50 μl), the volume of the mitochondrial sample was measured, and 1.5X volume of hydrochloric acid-acetone (5% 12 M hydrochloric acid, 95% acetone) was added to extract heme. HPLC was performed as described previously (30). The approximate percentage of each heme relative to the total amount of extractable hemes was calculated for each sample using the area under each heme peak in the chromatogram.

### Immunoblotting

BN-PAGE separation of mitochondrial protein complexes solubilized with 1% digitonin was performed as described (38, 57) using 5 to 13% gradient polyacrylamide gels (self-made) or 3 to 12% gradient polyacrylamide gels (Life Technologies). SDS-PAGE was performed using Any kD Mini-PROTEAN TGX gels (BioRad) or self-made 12% polyacrylamide gels. Proteins and protein complexes separated by SDS-PAGE and BN-PAGE, respectively, were transferred to nitrocellulose or PVDF membranes and blocked in 5% nonfat milk in PBS (with or without Tween-20). Membranes were incubated in the indicated primary antibodies and goat anti-mouse or anti-rabbit horseradish peroxidase-coupled secondary antibodies from Santa Cruz Biotechnology (sc-2005 and sc-2030), Jackson ImmunoResearch Laboratories (115–035–068), or Cell Signaling Technology (7074). Protein bands were visualized by incubation of membranes with chemiluminescence reagents (Millipore and Thermo Fisher Scientific), and images were captured with an Amersham Imager 600 (GE Healthcare) or by exposure to X-ray film (Thomas Scientific Inc.). The following primary antibodies were used in this study: mouse anti-Cox1 (ab110270, Abcam); mouse anti-Cox2 (ab110271, Abcam); mouse anti-Cox3 (ab110259, Abcam); mouse anti-Porin (459,500, Invitrogen); mouse anti-c-Myc (11,667,149,001, Roche Diagnostics); rabbit anti-Aco1 (a gift from Dr Roland Lill), rabbit anti-Aac2 (a gift from Dr Carla Koehler), rabbit anti-FLAG (F7425, Sigma-Aldrich); rabbit anti-Atp2 (reactive toward the Atp2 β subunit of F_1_F_0_ ATPase) kindly provided by Dr Alexander Tzagoloff; and rabbit anti-Cyt1, rabbit anti-Sdh2, and mouse anti-Rip1 kindly provided by Dr Dennis Winge. Antibodies were tested for reliability to assure specificity of detection at expected migration distances. For all immunoblots, each membrane was stripped and reprobed to detect further proteins. Western blot quantification was performed in ImageJ (58). When performing band quantifications of the levels of Cox15-FLAG and Cox15(G95R)-FLAG proteins from immunoblots, the anti-FLAG signal in each lane was divided by the signal for the loading control (either anti-Por1 or anti-Atp2) in the same lane for normalization. Statistical significance was determined by *t* test using the means of the technical replicates performed on each biological replicate (n = 3 biological replicates per sample).

### Membrane extractability and subcellular fractionations

The localization of Cox15, its mutant variants, and relevant control proteins to the mitochondrial membranes was analyzed by sodium carbonate extraction as previously described (59).

### qPCR analysis

qPCR was carried out as before (60), using an RNA purification kit (MasterPure Yeast RNA purification kit from Research Technologies) and a complementary DNA synthesis kit (Bio-Rad). The samples were analyzed in triplicate and the values normalized to β-actin expression levels. The values were calculated using the ΔΔC_T_ method. Statistical significance was determined by one-way ANOVA with Tukey HSD *post hoc* test, using the means of the technical replicates performed on each biological replicate (n = 5 biological replicates per sample).

### Protein modeling and bioinformatics

Amino acid sequence alignment was performed with use of the constraint-based multiple alignment tool (COBALT, U.S. National Library of Medicine's National Center for Biotechnology Information). Percent identity between Cox15 homologs was determined using BLAST (U.S. National Library of Medicine's National Center for Biotechnology Information). The Phyre2 protein fold recognition server (61) was used for one-to-one threading of the yeast Cox15 protein sequence onto the PDB 6A2J crystal structure of heme *a* synthase from *B. subtilis* (32). The yeast protein sequence used for this analysis lacked the first 65 amino acid residues, as they were predicted by MitoProt II (62) to comprise the mitochondrial targeting sequence, which is removed upon mitochondrial import. PyMOL (Schrödinger) was used to create the protein structure images.

## Data availability

All data in this article are available upon request to the corresponding authors (foxjl@cofc.edu or okhalimonchuk2@unl.edu).

## Supporting information

This article contains supporting information.

## Conflict of interest

The authors declare that they do not have any conflicts of interest with the contents of this article.
