# Supplementary material for: Molecular analysis of a new patient COX15 mutation provides insight into the etiology of fatal infantile cardioencephalopathy
Source: J Biol Chem. 2026 Apr 17;302(6):111475. doi: 10.1016/j.jbc.2026.111475 (PMC13213658; doi:10.1016/j.jbc.2026.111475)
Supplement: Supporting Information [file mmc1.docx]

**Molecular analysis of a new patient COX15 mutation provides insight into the etiology of fatal infantile cardioencephalopathy**

Jayda A. Carroll-Deaton, Iryna Bohovych, Faith T. Emetu, Jonathan V. Dietz, Elise D. Rivett, Elinor Stanley, Eric L. Hegg, Jennifer L. Fox, Oleh Khalimonchuk

Material included as Supporting Information: 1) Growth curves and 2) growth tests and Western blots using *cox15*Δ cells transformed with YCp plasmids bearing Cox15 or the G95R variant under the control of its native promoter


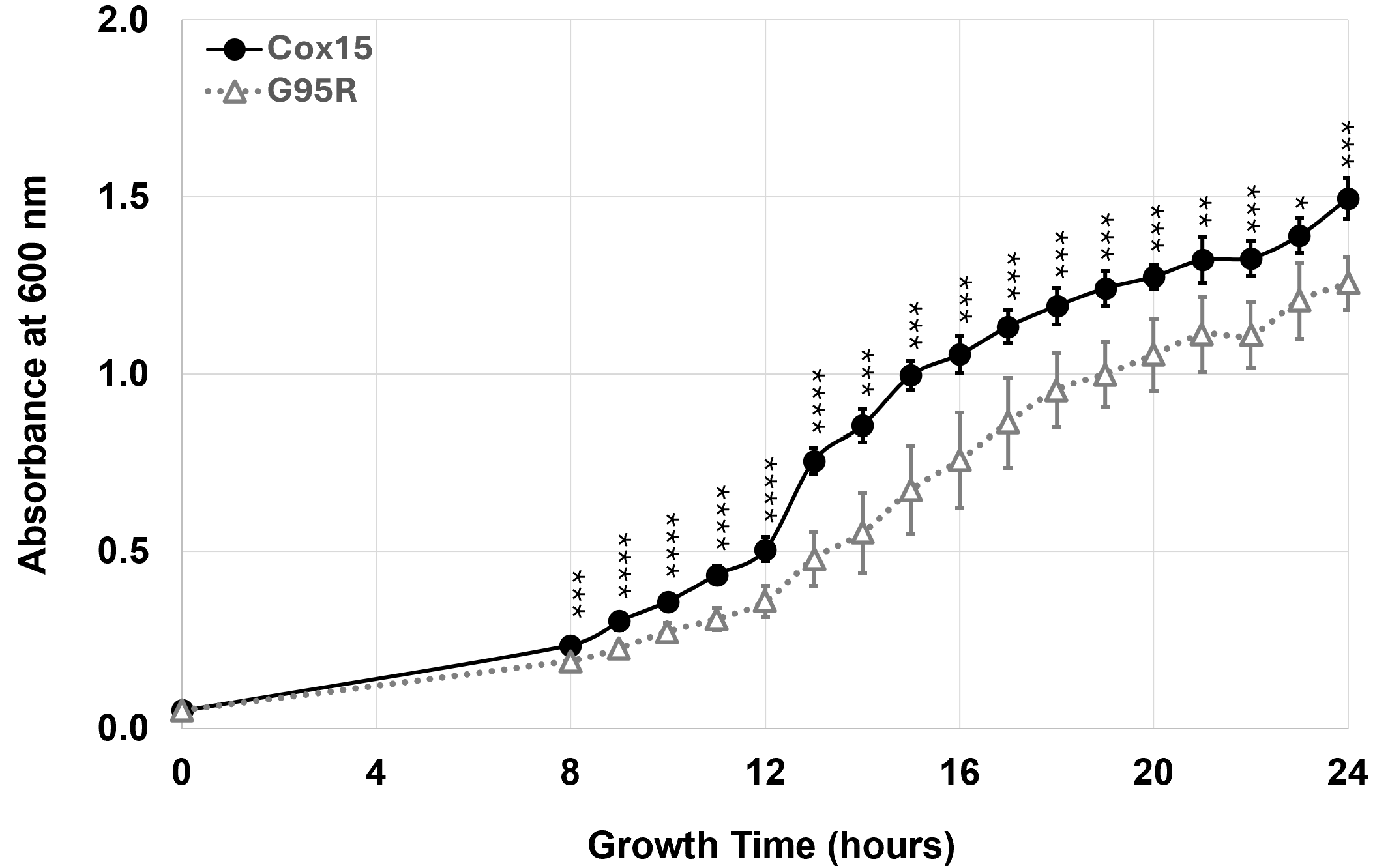


**Figure S1. Cellular growth of *cox15*Δ expressing the wild type or the G95R variant of Cox15 on non-repressive carbon source medium that permits respiration.** Cells were transformed with plasmids expressing either Cox15-FLAG (Cox15) or COX15(G95R)-FLAG (G95R) from the *MET25* promoter. Single colonies were inoculated in 5 ml of selective synthetic medium with 2% glucose and grown overnight at 30°C. Cells were then collected via gentle centrifugation at 3000 rpm for 3 min., washed with fresh synthetic medium containing 2% galactose and 0.1% glucose, resuspended in the same medium, and inoculated into 50 ml of pre-warmed (30°C) selective synthetic medium with 2% galactose and 0.1% glucose at a starting A_600_ of ~0.05. After 8 hours, the cellular growth was measured hourly for the duration of the next 16 hours. The growth curves represent the means from four biological replicates; the error bars reflect standard deviation. Statistical significance was calculated using non-paired t-test, with p values *<0.05, **<0.01, ***<0.005, and ****<0.001.







**Figure S2. Respiratory growth test** **at 30°C of *cox15*∆ cells expressing YCp-plasmid-borne WT Cox15-FLAG (YCp-*COX15*), the G95R Cox15-FLAG variant (YCp-G95R), or vector control [vector(YCp)] from the *COX15* promoter, along with WT cells.** Cells were cultured overnight in 2% glucose-containing synthetic medium lacking uracil for plasmid selection of the transformants or similar medium containing uracil for WT. Aqueous solutions of these cells normalized to A_600_ = 1 were serially diluted onto synthetic medium plates containing either 2% glucose or 2% glycerol/lactate (Gly./Lac.), with (+BPS) or without 100 μM bathophenanthroline disulfonate. Results are shown for one experiment, representative of three biological replicates.





**Figure S3. Immunoblots of mitochondrial proteins from *cox15*∆ cells expressing YCp-plasmid-borne WT Cox15-FLAG (YCp-*COX15*) or the G95R Cox15-FLAG variant (YCp-G95R) from the *COX15* promoter, compared to YEp-plasmid-borne versions of WT Cox15-FLAG (YEp-*COX15*) and the G95R Cox15-FLAG variant (YEp-G95R) from the *MET25* promoter, along with WT cells.** Cells were cultured in selective synthetic medium with 2% glucose. Immunoblotting was performed using anti-FLAG antibody (including both a shorter exposure and an extended exposure), and Ponceau S staining shows protein in each lane. The positions of molecular mass markers are indicated on the left side of the immunoblots. Results are shown for one experiment, representative of three independent experiments (biological replicates).
